# Return to work with fatigue after stroke: A complex occupational adaptation process

**DOI:** 10.1080/11038128.2026.2613621

**Published:** 2026-01-09

**Authors:** Jessica Vollertsen, Mathilda Björk, Anna-Karin Norlin, Elin Ekbladh

**Affiliations:** 1 Department of Rehabilitation, and Department of Health, Medicine and Caring Sciences, Linköping University, Motala, Sweden; 2 and Department of Health, Pain and Rehabilitation Center, Medicine and Caring Sciences, Linköping University, Linköping, Sweden; 3 Department of Health, Medicine and Caring Sciences, Linköping University, Linköping, Sweden

**Keywords:** everyday life, impairment, life after stroke, occupational identity, rehabilitation, sustainable return to work, Worker role interview

## Abstract

**Background:**

Return to work after stroke is a key goal in rehabilitation for people of working age. However, post-stroke fatigue is a common and complex symptom, and how it affects sustainable return-to-work and everyday life remains insufficiently explored.

**Aims/Objectives:**

To describe how persons of working age who experience fatigue after stroke perceive their prerequisites for sustainable return-to-work in the context of everyday life.

**Material and Methods:**

Forty-eight working-age individuals with stroke participated. Data were collected *via* the Worker Role Interview (WRI) and a survey. WRI data were analysed using qualitative abductive content analysis and descriptive statistical analysis were conducted.

**Results:**

Upon returning to work, participants found themselves in an everyday life where fatigue affected their work ability. The process was marked by uncertainty, ongoing challenges handling work demands under reduced capacity, and inconsistent support. The stroke marked the beginning of a complex, emotionally charged process as participants sought to navigate their new self and redefine their occupational identity.

**Conclusions/significance:**

Sustainable return-to-work when experiencing post-stroke fatigue requires person-centred rehabilitation that integrates work and everyday life. Flexible work environments are essential, along with close employer collaboration to ensure understanding of the work situation.

## Introduction

Work is a central and valued occupation, and studies have shown that return to work after stroke is beneficial for health, quality of life, autonomy and perceived participation [1,2] and thus an important goal for rehabilitation for people in working age [3]. However, in some cases, returning to work after a stroke can negatively impact a person’s well-being, contributing to a gradual decline in health and reduced energy for activities outside of work [4,5].

Despite regaining a high level of physical recovery after a stroke, people encounter difficulties in reintegrating into the workforce and maintaining employment [6,7]. Post-stroke fatigue has been identified as a challenge to return-to-work for both men and women [8,9], and one of the most challenging impairments to manage in everyday life after a stroke [10]. Post-stroke fatigue is a multifaceted symptom, likely influenced by biological, psychosocial, and behavioural factors [11]. However, its underlying pathophysiology remains poorly understood [12]. Fatigue can manifest in various forms or dimensions, including cognitive (or mental), emotional, physical, and stress-related aspects [13], and can impair cognitive functions such as attention and information processing speed [14], which are crucial for managing daily activities. It often arises from cognitively demanding tasks or environments, and its effects become more pronounced during sustained mental effort or in the presence of intense sensory stimulation [0014–0016]. In this article, the term ‘fatigue’ refers specifically to post-stroke fatigue with no limitations regarding dimensions. Fatigue levels may fluctuate throughout the day, influenced by both diurnal rhythms and the intensity and nature of ongoing activities [16]. It is also a major contributor to limitations in performing complex tasks, within work and household responsibilities [17].

Since people of working age often engage in occupations that require cognitive skills such as information processing, multitasking, and sustained attention in social settings, it is crucial to understand how fatigue impacts everyday life, particularly in relation to achieving a sustainable return-to-work after stroke. Sustainable return-to-work is a multifaceted concept that has been primarily explored in studies focusing on mental health or musculoskeletal disorders [18]. While definitions vary, sustainable return-to-work generally refers to a stable and continuous reintegration into the workforce [18,19], supported by a holistic approach that takes into account personal, social, and organizational factors [19]. Beekman [20] argues that return-to-work should be viewed as a process consisting of phases, transitions, and potential relapses, and that studies aiming to describe the return-to-work process often fail to capture the full complexity and diversity of return-to-work experiences, particularly the role of contextual factors [20]. The findings of Strømstad et al. [21] support this perspective by demonstrating that barriers to return-to-work are multifaceted and extend beyond individual health limitations and performance capacity. They also encompass components described within the Model of Human Occupation (MOHO), including volition, habituation, and environmental influences. Previous studies [22,23] have likewise shown that factors related to these components can predict whether individuals on sick leave due to mental or somatic conditions return to work within two years, underscoring the importance of considering motivation, role patterns, routines, and contextual conditions when evaluating sustainable return-to-work. However, much of the earlier research on return-to-work after stroke has primarily focused on the binary outcome of whether individuals returned to work, while only a limited number of studies have examined the sustainability of this return over time [24]. Wassenius et al. [25] emphasizes the importance of viewing return to work not as a singular outcome but as an interconnected part of everyday life after stroke, extending beyond the return to work. This perspective is particularly relevant given that having a stroke may lead to difficulties in managing various aspects of daily life, -making it challenging to manage work, family responsibilities, and household tasks [26]. However, the influence of fatigue on sustainable return-to-work and its broader impact on overall life remains insufficiently explored. Therefore, this study aims to describe how persons of working age who experience fatigue after a stroke perceive their prerequisites for sustainable return-to-work in the context of everyday life.

## Material and methods

### Design

This study employed a qualitative design, using abductive content analysis as methodological approach [27], complemented by descriptive quantitative data. We adhered to the Standards for Reporting Qualitative Research (SRQR) when reporting methods and findings [28]. The study was approved by the Swedish Ethical Review Authority (Dnr: 2020-01200).

### Inclusion criteria for participants

The inclusion criteria required participants to be between 18 and 63 years of age, to have had a stroke within the past five years, to be experiencing fatigue, and to have been gainfully employed at the time of stroke onset.

### Data collection

#### Worker role interview (WRI)

The Swedish version of the Worker role interview (WRI) [29] was used for data collection. The WRI is an assessment tool designed to identify psychosocial and environmental factors that influence an individual’s ability to remain in or return to work following injury or illness. It consists of a semi-structured interview and a therapist-administered four-point rating scale used to evaluate how each of the 16 items affects the client’s work ability. Each item is rated on a scale from 1 to 4, where 1 indicates that the item strongly interferes with returning to work, 2 that it interferes, 3 that it supports, and 4 that it strongly supports return to work. If an item is not applicable, it is marked (NA). In addition, the interviewer records qualitative comments to explain the chosen rating and describe how each item relates to the individual’s specific work ability.

The WRI is theoretically grounded in the MOHO [30], which conceptualizes human occupation through four interacting concepts: volition, habituation, performance capacity, and environmental influences. Within the WRI, these concepts are operationalized through the components personal causation, values, interests, roles, habits, and the physical and social environment, the components are further explained in Table 1 and the items related to each component in WRI are presented in Table 3. Building on this conceptual foundation, the WRI does not assess sustainability in return-to-work as an outcome; instead, it identifies psychosocial and environmental prerequisites that shape a person’s work potential in relation to occupational participation. These prerequisites, emphasized in MOHO as central determinants of occupational participation over time, are highly relevant for understanding conditions that support a sustainable return-to-work for individuals experiencing post-stroke fatigue.

**Table 1:** Example of the analysis process.

Key components in the WRI	Meaning unit	Subcategory	Category
*Personal causation,* i.e. sense of competence and effectiveness	I think I’ll have a hard time limiting myself	Altered capacity a challenge for a sustainable return to work	Re-evaluating work ability while experiencing fatigue
*Values,* i.e. images of what is important and meaningful in life, what is worth spending time on	The job becomes secondary. Now I’ve come to terms with it	Evolving identity – from loss to redefinition	Redefining identity in the context of everyday life
*Interests,* the enjoyment and stimuli one finds inside and outside work	Exercise, but not in the same way as before	Evolving identity – from loss to redefinition	Redefining identity in the context of everyday life
*Roles,* i.e. positions within social groups, shape identity and carry obligations and expectations for certain behaviours	Been a fixer at work, took a while for them [co-workers] to realize that I couldn’t help anymore	Fulfilling personal and others’ expectations in everyday responsibilities	Redefining identity in the context of everyday life
*Habits,* i.e. patterns of doings, refer both to the style of execution and to the way routines are structured and organized over time	Removed all the extras, kept everything simple	Structure and recovery in the midst of fatigue	Re-evaluating work ability while experiencing fatigue
*Environment,* i.e. the perceived opportunities, resources, demands, and constraints offered by the physical, social, and occupational related aspects of the context	I wouldn’t be able to get through the doors. The sounds and lights in the factory are overwhelming	Altered capacity a challenge for a sustainable return to work	Re-evaluating work ability while experiencing fatigue

The first version of the WRI was developed in the USA in 1991 and was later translated, culturally adapted, and then tested for use in a Swedish context [31]. The Swedish version has been evaluated for clinical utility and was considered effective, easy to use, and relevant by both occupational therapists and clients in vocational rehabilitation [32]. In addition, the WRI has shown predictive validity in identifying factors relevant to sustainable return to work following sick leave [22,23].

#### Questionnaire

A questionnaire was used to collect demographics—e.g. age, gender, years since stroke onset, work status, employment, education, housing, and social status. Furthermore, the questionnaire included: The Mental Fatigue Scale (MFS) [33] and the Hospital Anxiety and Depression Scale (HAD) [34], with the objective of describing participants’ characteristics. An online survey was created using the survey tool Survey & Report, along with a paper-based version. The MFS [33] is a self-assessment form designed to measure and assess the subjective experience of mental fatigue. A total of 15 questions are rated on a scale ranging from 0 to 3, allowing participants the flexibility to provide ratings in between (e.g. 0.5, 1.5, 2.5). Out of these, the 14 questions are summed up to produce a total score between 0 and 42, with a cut-off score of 10.5 indicating symptoms of mental fatigue. The score indicates three different levels of mental fatigue severity: mild (10.5–14.5), moderate (15–20) and severe (20.5–42) [35]. The MFS has shown good reliability and sensitivity in distinguishing mental fatigue, and the questions included in the scale have been found to have an adequate internal consistency [33]. The Hospital Anxiety and Depression Scale (HAD) consists of two subscales, 7 items measuring anxiety and 7 items measuring depression with a total score 0–21 for each scale [34]. The use of a total score with a cut-off of ≥8 is applicable for screening depression and anxiety in the later post-stroke phase [36].

### Recruitment and data collection procedure

Participants were recruited on seven occasions between 2020 and 2024 through an advertisement posted in three Facebook groups targeting individuals who had experienced a stroke. The advertisement included a brief description of the study, contact information to the first author (JV) and last author (EE), and a link to a digital interest registration form. In the form, potential participants received written study information, and they could also indicate whether they preferred to complete the questionnaire online or receive it by post. It was also possible to decline participation. Those who selected the online option were directed straight to the web-based questionnaire, while those who chose the paper format were asked to provide their name and postal address. Regardless of format, all potential participants received written study information, the questionnaire, and the option to provide informed consent to participate in the survey and/or be contacted for an interview.

A total of 59 persons gave their consent for an interview. Two persons were left out due to not meeting the inclusion criteria as they had had strokes more than five years prior to the study’s onset. The first author contacted 57 persons by mail or phone based on their stated preferences in the questionnaire. One reminder mail or phone call was sent out. In the event of an unanswered phone call, a message was read onto the answering machine. After a second unanswered phone call, a text message was also sent as a reminder. Nine persons could not be reached and were therefore considered non-responding. The 48 people who were reached agreed to be interviewed and an interview appointment was booked within two weeks. To ensure confidentiality, each participant was assigned a unique code number, which was used throughout the data handling and analysis process.

The first author (JV), an occupational therapist with over 20 years of clinical experience in stroke rehabilitation, conducted the WRI interviews. The interviews were conducted over telephone and lasted 60 to 75 min. Throughout the interview, the interviewer took notes relevant to the content of the WRI assessment. Immediately after the end of the interview, the rating form was completed. For each item, the interviewer conceptualized the participant’s unique situation in relation to the specific item definition and its theoretical framework in MOHO. Accordingly, the written comments on the rating form included direct statements as well as synthesized summaries informed by the interviewer’s conceptual understanding.

### Data analysis

Descriptive statistics were used to analyse the questionnaire responses and WRI item ratings. Numbers, percentages, means, and standard deviations (SD) described the participants’ demographics and characteristics. To describe whether the content of the WRI was perceived as supportive or interfering with return-to-work at a group level, percentages, medians, and interquartile ranges were used.

The written comments in relation to the conceptual understanding of each item on the WRI rating forms were analysed using abductive qualitative content analysis. This approach can be used to achieve a comprehensive understanding and involves an iterative movement between deductive and inductive reasoning [27]. The analysis process began with a structured deductive procedure guided by a directed approach [37]. Directed content analysis uses key concepts from existing theory as initial coding categories. In this study, the six key concepts—personal causation, values, interests, roles, habits, and environment in the MOHO and provided by the WRI—were used as the analytical matrix. The first author (JV) independently conducted a deductive analysis by coding the written comments using the predetermined categories. During this phase, the categorization of written comments was validated against the theoretical framework to confirm their alignment with the intended key concepts. Once data had been deductively organized according to the key theoretical concepts, the analysis proceeded inductively. The first (JV) and the last author (EE) engaged with the material through repeated readings to gain familiarity with the content and its overall meaning and made notes of initial impressions and reflections [37]. Subsequently, the authors JV and EE engaged in a reflexive discussion to identify patterns and categories [27]. All authors were actively involved in the final discussions, which led to a consensus on the categories representing the study’s results, see Table 1 for an example of the analysis process. The authors represent diverse professional backgrounds: three occupational therapists (JV, MB, EE), and one physician (AKN), all with expertise in vocational rehabilitation and fatigue. The last author (EE), skilled in MOHO and the WRI, contributed to ensuring a consistent understanding of the key theoretical concepts among researchers. None of the authors had any prior relationship with the participants involved in the study.

## Results

### Demographics and characteristics of the participants

A total of 48 individuals participated in the study, 37 of whom were women. Participant demographics and characteristics are presented in Table 2. The mean age at stroke onset was 48 years (SD =7) for women and 51 years (SD =5) for men. Interviews were conducted one to five years post-stroke, most commonly after one year (*n* = 21; 44%). At the time of the interview, 63% (*n* = 30) lived with another adult, and of these, 26 (54%) had children living at home. Among the 18 (38%) participants in single-adult households, 9 (19%) were single parents. Regarding employment, 96% of the participants were employed. Of these, 73% (*n* = 35) were working to some extent: 15% (*n* = 7) were working full-time, 58% (*n* = 28) were working part-time (25%–75%), while 23% (*n* = 11) were on full-time sick leave.

**Table 2:** Participant demographics and characteristics (*n* = 48).

Demographics and characteristics	Values
Total, n	48
Female sex, n (%)	37 (77)
Age at stroke year (mean + SD)	49 + 7
Years since stroke (mean + median)	2.5 + 2.0
Cohabiting, n (%)	30 (63)
Participants with children living at home, n (%)	26 (54)
Age of the children living at home, (n)	
≤ 12	14
13–18	19
19 ≥	10
Educational level, n (%) (n = 47)	
Compulsory school	0
High school	17 (36)
University	30 (64)
Employment, n (%) (n = 47)	
Permanent employment	41 (87)
Temporarily employed	4
Unemployed	2
Working status, n (%) (n = 48)	
Working 100 %	7 (15)
Working 25–75 %	28 (58)
Sick leave 100 %	11 (23)
Sickness compensation 100 % (early retirement)	2 (4)
Mental Fatigue Scale, n (%) (n = 43)	
No mental fatigue (0–10)	5 (11.5)
Mild (10.5–14.5)	5 (11.5)
Moderate (15–20)	17 (40)
Severe (20.5–42)	16 (37)
Hospital Anxiety and Depression Scale, n (%)	
No indication of depression, (score < 8) (n = 40)	32 (80)
No indication of anxiety, (score < 8) (n = 41)	24 (59)

According to the Mental Fatigue Scale (MFS), all but five participants (89%) reported mental fatigue to some extent, most at moderate to severe levels. Based on the Hospital Anxiety and Depression Scale (HADS), the majority did not show clinically significant symptoms of depression (80%) or anxiety (59%).

### Participants’ prerequisites for sustainable return to work based on WRI item ratings

The WRI items most frequently rated as supportive of returning to or remaining at work were ‘Enjoys work’ (90%; *n* = 43), ‘Appraises work expectations’ (87%, *n* = 42) and ‘Commitment to work’ (83%, *n* = 40). The items most rated as interfering with returning to or remaining at work were ‘Perceptions of the work environment’ (67%, *n* = 32), ‘Expectations of job success (60%; *n* = 29) and ‘Pursues interests’ (58%; *n* = 28). An overview of all WRI item ratings is presented in Table 3.

**Table 3:** Ratings of the 16 WRI items among participants (*n* = 48).

WRI item	Interferes **rating 1 & 2** n, (%)	Supports **rating 3 & 4** n, (%)	Median	Interquartile range
*Personal causation*				
1. Assesses abilities and limitations	15 (31)	33 (69)	4.0	2.00 − 4.00
2. Expectations of job success	29 (60)	19 (40)	2.0	2.00 − 4.00
3. Take responsibility	10 (21)	38 (79)	3.0	3.00 − 4.00
*Values*				
4. Commitment to work	8 (17)	40 (83)	4.0	3.00 − 4.00
5. Work-related goals	12 (25)	36 (75)	3.0	2.25 − 4.00
*Interests*				
6. Enjoys work	5 (10)	43 (90)	3.0	3.00 − 4.00
7. Pursues interests	28 (58)	20 (42)	2.0	1.25 − 3.00
*Roles*				
8. Appraises work expectations	6 (13)	42 (87)	4.0	4.00 − 4.00
9. Influence of other roles	24 (50)	24 (50)	2.5	2.00 − 4.00
*Habits*				
10. Work habits**	16 (35)	30 (65)	3.0	2.00 − 4.00
11. Daily routines	15 (31)	33 (69)	3.0	2.00 − 3.00
12. Adapts routines to minimize difficulties	18 (38)	30 (62)	3.0	2.00 − 4.00
*Environment*				
13. Perception of the work setting	32 (67)	16 (33)	2.0	1.00 − 3.00
14. Perception of family and peers	24 (50)	24 (50)	2.5	2.00 − 3.00
15. Perception of boss*	20 (43)	27 (57)	3.0	1.00 − 4.00
16. Perception of co-workers*	15 (32)	32 (68)	3.0	2.00 − 4.00

Note.

* representing that rating was not applicable for one participant.

** representing that rating was not applicable for two participants.

### Perceived prerequisites for sustainable return-to-work based on written comments on WRI items

**Figure 1:**
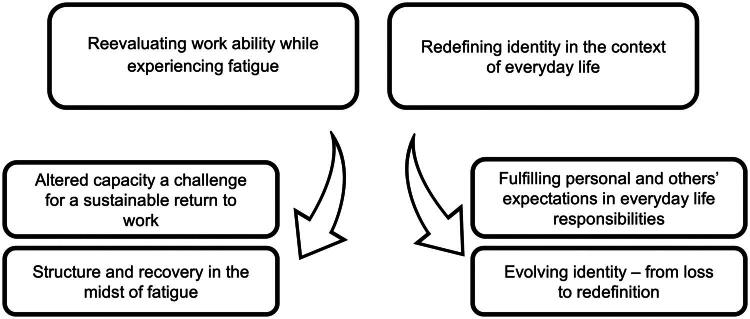
Categories and subcategories developed during the analysis.

The abductive content analysis resulted in two categories: *re-evaluating work ability while experiencing fatigue* and *redefining identity in the context of everyday life*. The categories and associated subcategories are seen in Figure 1.

#### Re-evaluating work ability while experiencing fatigue

The participants described an everyday life in which fatigue had altered their capacity, challenging their sense of competence and effectiveness in work and other occupations. Recognizing how these changes affected their occupational performance across everyday life was closely tied to an increased self-awareness of their needs for a sustainable work situation. In this process, participants needed to develop and establish habits and routines that supported occupational performance both at work and in life outside work. The material revealed the following two subcategories that illustrate these intertwined challenges: *altered capacity a challenge for a sustainable return to work* and *structure and recovery in the midst of fatigue.*

##### Altered capacity a challenge for a sustainable return to work

Participants described fatigue as an unpredictable and persistent alteration in their capacity, often manifesting through memory difficulties, challenges with concentration and multitasking, and increased sensitivity to stress. This unpredictability created a need to understand when, why, and how fatigue occurred, both in everyday life and in relation to work. Participants emphasized the importance of recognizing early signs of fatigue to prevent setbacks during the return-to-work process. Ignoring these signals often led to physical symptoms such as headaches, migraines, or overwhelming exhaustion lasting from a day to a week. These experiences reflect disruptions in personal causation, which encompasses both the appraisal of one’s abilities and the confidence to use them effectively.

The altered capacity was not always visible to others, which made it difficult for participants to gain recognition and understanding from supervisors, co-workers, and external contacts. The social work environment was viewed as central to a sustainable return to work. Supportive supervisors who actively sought to understand the nature and impact of participants’ altered capacity were crucial. However, frequent changes in leadership created uncertainty, as new supervisors often lacked awareness of participants’ previous work ability and the effects of their condition. *‘At the time of my stroke, I had a supervisor who took responsibility and helped me with adapted work tasks. When he left the next supervisor removed that possibility for me’.* While technical aids, such as earplugs or quiet keyboards, were generally easy to obtain, flexible adjustments to tasks, schedules, or workspace were often less available. Participants did not primarily struggle to express these needs, but rather to have them understood and regarded as legitimate by supervisors. Sensitivities to light and noise were seen as concrete and valid, making these needs easier to acknowledge, whereas task-related needs were frequently misunderstood or dismissed, reducing the likelihood of accommodation.

Support from co-workers was described as especially valuable when close working relationships had been established, as familiarity enabled them to notice subtle signs of fatigue and help with task adjustments. In contrast, interactions with external contacts, such as customers, clients, or students, were particularly demanding, as these individuals often assumed that participants had fully recovered. As one participant explained, ‘*It doesn’t work for me to be at the register or on the floor because customers still see me as the old Maria’.* To cope, participants often sought out tasks with reduced social demands. The participants’ experiences reflect efforts to take responsibility for achieving a manageable work situation. However, their ability to do so was constrained by limited external control over the work conditions often due to supervisors insufficient understanding of their needs.

Gradual return-to-work and work training were seen as valuable for understanding changed abilities, limitations, and the impact of fatigue on occupational participation. Many participants expressed a continued need for vocational rehabilitation even after resuming full-time work, as they managed the persistent impact of altered capacity. However, some experienced an abrupt withdrawal of support upon reaching full-time hours: ‘*at 100%, they let go, and that’s when all the questions came’.* Stroke-specific rehabilitation was helpful in supporting communication about the invisible and fluctuating nature of the impairment, although access to such programs was not always available. Some participants also described external pressure from employers or the Social Insurance Agency to increase work hours prematurely, often leading to setbacks and renewed sick leave: ‘*I have to prove that it doesn’t work by feeling unwell. I’ve been forced to crash 3–4 times’.* This pressure contributed to feelings of lost control and reduced agency in the return-to-work process. At the same time, support from the Social Insurance Agency or unions was highlighted as crucial, particularly when participants felt they were perceived as less productive or burdensome by supervisors or co-workers. These experiences were closely linked to the unpredictable nature of fatigue and the uncertain trajectory of recovery; factors participants felt were difficult for employers to fully understand. Fatigue undermined participants’ belief in their ability to sustain work in everyday life, negatively affecting their motivation.

##### Structure and recovery in the midst of fatigue

Participants highlighted how daily routines outside work influenced their work ability and emphasized the interaction between work and life outside of work. A lack of structure in everyday life often led to unpredictability and negative spill-over effects at work. Fatigue left no room for unexpected events, as seemingly minor disruptions, such as a child missing the school bus or misplaced car keys, could drain participants mentally and impact an entire workday. Routines were described as essential for maintaining control and for conserving energy. Participants described strategies such as planning outfits the night before, using calendars or reminders, and maintaining an organized home. As one participant expressed: *‘a cluttered mind requires order and tidiness around it’*. However, many struggled to implement routines and maintain them, as their fatigue consistently interfered with their efforts.

To cope with fatigue, all participants emphasized the importance of incorporating rest and recovery into their daily routines. They adopted a proactive approach, carefully planning and scheduling breaks both during and outside of work hours to sustain their energy levels. After a workday, rest was seen as essential, although the form and duration varied. Rest could include sleeping, lying in a dark room, reclining while reading, or taking a walk. Participants also emphasized the importance of integrating rest into leisure activities for example: ‘*I have a boat, which helps because I can lie down in it and rest whenever I need to’,* or bringing a yoga mat on a walk in the woods to allow for restorative pauses. To navigate workplace expectations while addressing their need for recovery, several participants developed strategies such as taking breaks in restrooms, a practice they perceived as discreet and socially accepted, and one thought was generally unquestioned by supervisors and co-workers. Additionally, some expressed appreciation for workplace adaptations accelerated by the COVID-19 pandemic, particularly the increased use of digital meetings, which facilitated remote work. The flexibility of working from home allowed participants to conserve energy more efficiently by eliminating commuting and allowed them to utilize designated rest areas within their home environment. Although the intention to manage fatigue through the establishment of routines and habits in everyday life and by integrating rest and recovery into everyday life was shared by all participants, their success in doing so varied.

#### Redefining identity in the context of everyday life

The stroke marked the beginning of a complex and emotionally charged process in which participants sought to navigate their new self and redefine their occupational identity. As they sought to sustain their participation in work alongside the demands of everyday life, shifts occurred in the values and personal convictions that guided what they considered meaningful, as well as in the sense of obligation they felt towards themselves and others. Participants described how the limitations imposed by fatigue compelled them to reassess what aspects of work and everyday life they should prioritize, what they needed to let go of, and how these decisions shaped their evolving sense of who they were. The two subcategories illustrate different facets of this ongoing process: *fulfilling personal and others’ expectations in everyday responsibilities* and *evolving identity—from loss to redefinition.*

##### Fulfilling personal and others’ expectations in everyday responsibilities

Work was described as a valued occupation, deeply tied to a sense of duty, personal fulfilment, and financial stability. Many participants described that they had chosen careers based on a desire to help others, finding meaning in their work beyond external rewards. However, after experiencing fatigue, they had been forced to re-consider what they regarded as most important about their work. Some found new ways to derive meaning, while others saw work primarily as a financial necessity. For those with a strong occupational identity tied to performance at work, adjusting expectations was particularly difficult. One participant expressed, *‘It is extremely important to do a good job. It’s difficult to lower demands in an environment of high performers’.* Trying to maintain past performance levels often led to exhaustion. As participants reassessed the role of work in their lives, these internal reflections were often mirrored by their family members, who observed the toll fatigue was taking on them. Participants described how their family members were gaining a profound understanding of the pervasive effects of fatigue by witnessing its impact firsthand. However, this increased awareness also raised concerns among family members, particularly regarding the risk of another stroke. Many questioned whether participants should continue working full-time, or even remain in the workforce at all, after observing the consequences of their fatigue.

Participants struggled to maintain and fulfil expectations of vital roles beyond work. The new everyday life altered relationship dynamics between family members. Participants described how limited energy challenged their ability to care for their children in accordance with their own expectations and ideals of parenthood. One mother expressed, *‘I don’t have the energy to be a mom, it’s a lot of yogurt and porridge when work becomes too much’*, illustrating how fatigue challenged her ability to fulfil her parental role, including preparing proper meals. Those participants who were parents of adolescents described fatigue causing changes in their parental roles, as teenagers increasingly took on household responsibilities traditionally managed by parents. This transition was often framed as a mutual agreement, perceived as a natural progression in their children’s journey towards adulthood. These altered relationship dynamics were also described between spouses. Some participants emphasized the need for reduced working hours or stepping away from work entirely to experience a sense of fulfilment of participation in important roles beyond work. Participants expressed how fatigue limited their ability to engage in previously shared meaningful activities, activities that had once played a crucial role in maintaining and strengthening their relationships. While some expressed concerns about how long their spouse would tolerate these changes, others described a growing sense of closeness through increased emotional support.

##### Evolving identity—from loss to redefinition

To sustain work while managing fatigue after a stroke, participants made deliberate occupational choices. Some moved closer to work to make commuting easier, downsized from house to an apartment to reduce household maintenance, or abandoned plans for further education. These choices, although significant, were made after careful reflection, allowing participants to feel a sense of control over their decisions. In contrast, letting go of activities in everyday life was more emotionally challenging, as these changes were not proactive choices but necessary sacrifices made in everyday life due to limited energy. *‘What once fuelled me now drains me. Even friends and exercise feel impossible at 70–75% workload. This is sorrow’.* Many had to prioritize work, domestic responsibilities, and family, forcing them to give up previously enjoyed physical and social activities. This loss led to feelings of grief and a sense of disconnection from their former selves. However, participants described a shift over time from mourning to acceptance. One participant captured this transformation, stating, *‘This is the new me, Eva 2.0.’* Her words reflect both the loss of her former identity and the resilience of embracing a new reality shaped by her circumstances. This process involved discovering new ways to enjoy life despite fatigue, appreciating the small things in life, and creating a meaningful existence, all while striving for a sustainable return to work. For others, however, it was described as being about giving up work or being stuck in limbo, somewhere in between, simply trying to survive their life situation.

## Discussion

The results of this study describe prerequisites that shape the conditions for a sustainable return-to-work among persons of working age experiencing post-stroke fatigue. The WRI ratings show how motivational, lifestyle and environmental related factors interact to influence work participation, while the qualitative analysis revealed how these factors unfold in the unique persons everyday contexts. By integrating these perspectives, overall, this study shows that sustainability in return-to-work is an ongoing process of re-evaluating work ability and redefining identity in the new everyday life while experiencing fatigue.

The WRI ratings suggested that most participants found their work both enjoyable and stimulating. They derived a sense of importance and meaning from their work and clearly understood what was expected of them in their role as workers. However, the ratings also highlighted challenges related to diminished personal causation, where reduced sense of competence and effectiveness lowered participant’s confidence in their ability to return to work successfully, factors that have been shown to be important for return-to-work after sick leave [22,23]. The study findings suggest that, although participants valued work, their post-stroke capacity had shifted in ways that complicated their return-to-work trajectory. The qualitative content analysis adds further depth to this understanding by illustrating how these altered capacities extend beyond the workplace and into everyday life.

Participants struggled to manage responsibilities at work and at home. Fulfilling previously internalized roles required negotiating work as part of everyday life, adapting to changes in family life, and often prioritizing these demands over participation in leisure activities. Furthermore, demands in the workplace environment hindered the likelihood of accomplishing a manageable work situation. Thus, rather than representing parallel issues, the challenges in and outside work should be understood as integral to the return-to-work process itself. This aligns with previous findings showing that fatigue limit the ability to resume work and everyday activities to the same extent as prior to the stroke [17]. Further, it contributes to current knowledge by illustrating how life outside of work influences the return-to-work process and its outcomes, an area that has been increasingly acknowledged in recent studies [20,38,39]. Occupational identity emerged as a central and sometimes conflicting element. Many participants had defined themselves through a history of high work performance, making it difficult to reconcile current limitations with previous expectations. Due to MOHO, occupational identity reflects how individuals see themselves through past, present, and future engagement in meaningful activities. When this identity as a high performer is challenged by fatigue, acknowledging limitations may feel incompatible with one’s sense of self [30], potentially leading to exhaustion and recurrent sick leave. Although the majority did not self-report depression or anxiety, four out of ten still reported anxiety, suggesting an underlying worry about how life might unfold given uncertainty about their future roles and capacities. These findings emphasize that supporting sustainable return-to-work requires acknowledging the emotional and identity-related adaptations that accompany fatigue. Previous research similarly highlights that reconstructing meaning and re-engaging in everyday occupations are central to rebuilding a renewed sense of identity and wellbeing after stroke [40]. Such engagement also supports adaptation to post-stroke limitations, including fatigue [5,38,39,41,42]. From a clinical perspective, these insights underscore the importance of addressing these factors in practice, by facilitating meaningful activities, supporting identity reconstruction, and promoting strategies that enable participation in both work and everyday life despite fatigue. Hence, sustainable return-to-work involves more than adjusting to available personal energy; it is an emotionally charged process of occupational adaptation. This process entails challenges related to reshaping and reconstructing one’s occupational life [30,42].

Self-management strategies, such as rest, recovery, structure, and routines, were perceived as key to sustainable return-to-work. This result reflects previous research showing that routines and strategies can mitigate the impact of fatigue in everyday life [38,39]. Importantly, the findings also reveal a paradox; while structure and routine are essential for managing everyday life after stroke, fatigue itself makes it difficult to establish and maintain them. This emphasizes the complexity of achieving sustainability in returning to work. Gradually increasing work hours or participating in work training provided opportunities to gain insight into how fatigue affected the everyday lives of the participants, bridging the gap between planning and lived reality. This grading can be understood as exploratory phases during which individuals assess their abilities and limitations and begin developing strategies to engage in occupations within the constraints of an altered capacity, aiming to regain control over their everyday life [30]. Thus, interventions that focus on occupational participation can help individuals manage persistent symptoms, adapt to a changed sense of self, foster agency, and establish control in their everyday lives [43], aligning with priorities identified for life after stroke [44].

A supportive social work environment was described as central to facilitating a sustainable return to work. Participants emphasized that such support extended beyond physical adjustments, it required a nuanced understanding of the impact of fatigue on the individual, along with tailored and consistent assistance, even beyond the point of returning full-time. However, flexibility in tasks and schedules was often difficult to obtain, and frequent leadership changes forced participants to repeatedly explain their condition, undermining continuity and trust, factors that may reduce the likelihood of long-term return-to-work [45]. In contrast, co-workers with close working relationships played a key role in providing informal adjustments, recognizing subtle signs of fatigue, and offering support. Supervisors who actively engaged in understanding the nature and consequences of fatigue were also perceived as crucial. These relational forms of support helped participants manage fatigue at work and promoted sustainability, ultimately supporting participation in everyday life. These findings highlight the need for systematic, clear information about post-stroke fatigue [38,46], as increasing awareness among co-workers and supervisors can enable more appropriate support and accommodations [47]. Furthermore, future research should explore workplace-related factors in greater depth, as the findings indicate that essential conditions within the work environment significantly influence the sustainability of return-to-work after stroke. This aligns with previous research highlighting that workplace adaptations and close collaboration with managers and colleagues are crucial components of person-centred return-to-work programs, underscoring the complexity and importance of addressing environmental factors alongside individual needs to achieve sustainable return-to-work [48]. Addressing these factors is consistent with broader policy goals, as the results are in line with the Action Plan for Stroke in Europe 2018–2030 [45] and the Swedish Integrated Care reform [49], both of which emphasize person-centered, preventive, and long-term support. There is a need for further research into interventions that facilitate sustainable return-to-work, promote long-term health, and mitigate the risk of recurrent sick leave associated with fatigue.

## Methodological considerations

This study employed an abductive content analysis complemented by descriptive quantitative data. The trustworthiness of qualitative content analysis can be scrutinized by examining the different phases: preparation, organization, and reporting of results [50]. The use of the WRI, grounded in the conceptual model MOHO [30], and its aim of identifying the psychosocial and environmental factors influencing an individual’s ability to remain in or return to work, can be regarded as a strength. An additional strength of the study is the high participation rate, possibly due to the use of telephone interviews, which are suitable for individuals with exhaustion symptoms [51], and enabled nationwide recruitment without requiring travel. However, relying solely on Facebook groups may have excluded potential participants, as this recruitment method can limit sample diversity, given that marginalized individuals are often underrepresented or less active in such online communities [52]. This may limit the generalisability of the findings as the participants in the study may not reflect the broader population. The first interviews were performed during the COVID-19 pandemic in 2020. However, the data collection period was prolonged post-pandemic to capture different experiences from the participants. Although Sweden didn’t have a full lockdown, the pandemic affected how people interacted in society, as social interactions were limited. The abductive content analysis began with a deductive phase, initially conducted in collaboration between the first (JV) and the last authors (EE), and progressed to an inductive phase in which all authors were involved. An approach where one researcher leads the analysis and others critically review the process and categorization may enhance trustworthiness [50]. Furthermore, information power was strengthened by applying the well-established theoretical framework MOHO. Although rich dialogue occurred, the absence of audio recordings limited opportunities for verbatim analysis, potentially affecting nuance and depth. To compensate for this and ensure sufficient information power, a total of 48 interviews were conducted [53]. In reporting the results, direct quotations were used both to illustrate participants’ perspectives [54] and to enhance conformability [50].

## Conclusions

Sustainable return-to-work when experiencing post-stroke fatigue is a complex and emotionally charged process of occupational adaptation. Fatigue not only limits work ability but also challenges participation in home and leisure activities, undermining established roles, routines, and the individual’s sense of self. This study highlights the need for person-centered rehabilitation that supports the reconstruction of occupational identity, enables engagement in meaningful activities, and addresses the interdependence between work and everyday life.

Self-management of fatigue that supports recovery and rest, continuous individualized follow-up, and supportive work environments seem essential to sustaining long-term work participation and reducing the risk of recurrent sick leave. Rehabilitation professionals need to be aware of the paradox wherein fatigue impairs the ability to implement the very strategies—such as structure and routines—that are crucial for managing it.

Close collaboration with employers is vital to foster a nuanced understanding of post-stroke fatigue, ensure flexible, tailored support, and maintain continuity throughout the return-to-work process and beyond, as ongoing, adaptive support is often required.

## Generative artificial intelligence (AI)

The authors used ChatGPT (OpenAI) to assist with some language editing and improving readability. All content was reviewed and approved by the authors, who take full responsibility for the final manuscript.
